# Pitaya as a New Alternative Crop for Iberian Peninsula: Biology and Edaphoclimatic Requirements

**DOI:** 10.3390/plants12183212

**Published:** 2023-09-08

**Authors:** Ana Rita Trindade, Paulo Paiva, Vander Lacerda, Natália Marques, Luís Neto, Amílcar Duarte

**Affiliations:** 1 MED—Mediterranean Institute for Agriculture, Environment and Development and CHANGE—Global Change and Sustainability Institute, Faculdade de Ciências e Tecnologia, Universidade do Algarve (UAlg), Campus de Gambelas, 8005-139 Faro, Portugal; artrindade@ualg.pt (A.R.T.); vander.rocha@unesp.br (V.L.); lneto@ualg.pt (L.N.); 2 Instituto Federal de Educação, Ciência e Tecnologia do Triangulo Mineiro (IFTM), Uberaba 38064-790, Brazil; paulopaiva@iftm.edu.br; 3 Departamento de Produção Vegetal (Horticultura), Faculdade de Ciências Agronômicas, Universidade Estadual Paulista (UNESP), Botucatu 18610-034, Brazil; 4 CEOT—Centro de Eletrónica, Optoeletrónica e Telecomunicações, Faculdade de Ciências e Tecnologia, Edif. 8, Universidade do Algarve, Campus de Gambelas, 8005-139 Faro, Portugal; nmarques@ualg.pt

**Keywords:** Cactaceae, *Selenicereus*, dragon fruit, Europe, review

## Abstract

Pitaya is one of the fruit species whose demand has increased in recent years due to the numerous health benefits and lucrative price of the fruit and its by-products. In Europe, the Iberian Peninsula and other Mediterranean countries are the ones with favorable climatic conditions for its cultivation. This document describes much of the history of pitaya in the Iberian Peninsula and the difficulties related to its cultivation. A bibliographical survey was carried out on the culture of pitaya in the world, focusing on the edaphoclimatic requirements, and on the possibility of this becoming a consolidated crop in the Iberian Peninsula. The relatively low water requirement of pitaya makes this crop sustainable among crops that require irrigation. In addition, we provide a perspective for use and research of this emerging crop. There has been an exponential growth of scientific publications on pitaya in the last decade; however, much more needs to be researched to know how to increase productivity as well as the sensory quality of fruits in different regions. This sustainable crop is a good option to diversify fruit production in the Iberian Peninsula.

## 1. Introduction

The Mediterranean Basin and particularly the Iberian Peninsula are characterized by a diversified agriculture, with a predominance of vegetable and fruit crops, associated with the Mediterranean diet. Most of them were introduced in Iberian Peninsula over centuries. The Phoenicians and Greeks introduced the olive tree (*Olea europaea* L.) which today produces typical olive oil [1]. The carob tree (*Ceratonia siliqua* L.) reached the Iberian Peninsula with the Arab invasions [2,3]. Citrus (*Citrus* spp.) fruits were already known to the Romans who cultivated them throughout southern Europe, but it was only when Portuguese navigators discovered the sea route to India and China that the best sweet orange cultivars arrived, making this crop one of the most important in the Iberian Peninsula [4]. The introduction of new crops did not stop in these remote times. Some crops that are common today in the Iberian Peninsula were introduced or started to be cultivated less than a hundred years ago. The Japanese loquat (*Eriobotrya japonica* (Thunb.) Lindl.), which had been introduced as an ornamental in the 18th century, only began to be cultivated as a fruit crop in the 20th century [5]. Other species that began to be commercially cultivated in the 20th century were the mango (*Mangifera indica* L.), avocado (*Persea americana* Mill.) or Cape gooseberry (*Physalis peruviana* L.), although some had already been introduced a few centuries earlier. Even the native strawberry tree (*Arbutus unedo* L.) only came to be considered a crop at the end of the 20th century. In this incessant search for new crops to enrich Mediterranean agriculture and Mediterranean diet, pitaya has also started to be cultivated as a fruit crop in recent years.

Pitaya is native to tropical and subtropical rainforests, mainly Central America, and is an important crop in Brazil, Ecuador, Colombia, Costa Rica and Mexico [6]. Due to the growing demand around the world for this fruit, in recent decades the crop has spread to many countries. Vietnam and China are the world’s largest producers of pitaya. Other Asian countries, such as the Philippines, Thailand and Sri Lanka, also have significant production [7]. Pitaya is still in the phase of domestication, consequently demanding scientific and technical knowledge to support its cultivation. Production systems vary with production areas, from agroforestry systems in Latin America to greenhouse production in the USA (California) [8,9]. There has also been important progress in genetic improvement, mainly in Israel [10,11], but also in Brazil, United States and other countries. The introduction and growth of the area of pitaya cultivation in the Iberian Peninsula results, therefore, of the demand for new crops in this region of the globe and follows the world trend of expansion of crops whose fruit is associated with healthy diet.

The viability of this crop in the Iberian Peninsula was studied within the scope of the project “GO Fruta Dragão: validating the productive capacity of the red pitaya”. This review is somewhat the result of the information that has been gathered throughout this project, with some remarks on the challenges posed to the commercial production of pitaya in this region.

## 2. Current Pitaya Distribution in Iberian Peninsula

The distribution of pitaya cultivation in Europe is still limited, but it is gradually expanding in Spain, Portugal, Italy and Greece [12]. In Italy, the cultivation of pitaya was experimentally introduced by Dr. Ottavio Cacioppo in 1988 and today the plant is cultivated in the south of the country, mainly in the provinces of Sicily, Puglia, Calabria and Lazio [13,14]. It is also produced as an ornamental plant [12]. In Greece, pitaya is grown on the mainland (near Athens) and on the island of Rhodes, mainly by small producers [15,16].

**Figure 1:**
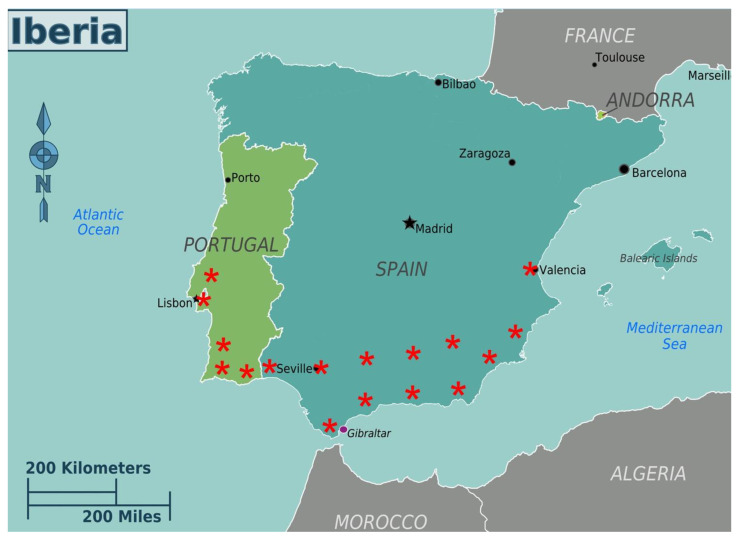
Map of the Iberian Peninsula highlighting the regions where pitaya is cultivated. * Indicates the regions with pitaya cultivation.

Spain is currently the largest pitaya producer in Europe with production concentrated in the southern region, in the Community of Andalusia. Pitaya began to be cultivated in the province of Huelva, in 2017, mainly in the municipalities of Lucena del Puerto, Almonte, Moguer, Palos de la Frontera, San Bartolomé de la Torre, Cartaya and Lepe. The area of this crop is growing in the province of Seville, mainly in the municipalities of Carmona, Dos Hermanas and Aznalcóllar and Guillena [17]. In other parts of the Andalusian community, such as Cádiz, Málaga and Almería, pitaya cultivation has seen great development in recent years (Figure 1).

In Portugal, pitaya cultivation is mainly located in the Algarve region, although there are plantations in Alentejo, Ribatejo and Extremadura [18]. In the Algarve, pitaya is cultivated mainly along the south coast or near the Guadiana and Arade rivers, mainly in the municipalities of Castro Marim, Vila Real de Santo António, Tavira, Olhão, Faro, Loulé, Albufeira and Silves [19]. Most plantations are in greenhouses. About half of the of the area is being grown hydroponically.

In the Iberian Peninsula, as in other southern European countries, pitaya is predominantly cultivated in small and medium-sized farms, often by individual producers interested in diversifying their crops or exploring new market opportunities. Pitaya has usually a high market value, an average of nine euros (€) per kilogram (kg) in the last years, due to high demand for its positive effects on health and low availability on the market, as production is small. Much of the production is sold in local markets, directly from the producer to the consumer, according to the European short agri-food circuit concept, avoiding long-distance transport and the consequent environmental impact and helping to stimulate the local economy.

## 3. Taxonomy and Botany

### 3.1. Family and Genera

Pitaya belongs to the Cactaceae family and the Cactoideae subfamily. The taxonomy of the Cactaceae family is not well defined. The botanical classification has undergone successive modifications and relatively recent publications adopt different botanical classifications. Cactoideae subfamily comprises epiphytic, hemiepiphyte and climbing cacti, but the genera within this subfamily are poorly defined and there is not a consensus among taxonomists. Recent studies, based on molecular markers, have shown that genus *Hylocereus* (A. Berger) Britton and Rose, which includes most cultivated pitayas, should be grouped in the genus *Selenicereus* (A. Berger) Britton and Rose [20]; however, prestigious researchers specializing in pitaya genetics continue to distinguish the two genera separately [11]. This has negative implications, as identical plants are referred to with different scientific names, confusing readers (researchers, agronomists and farmers).

**Figure 2:**
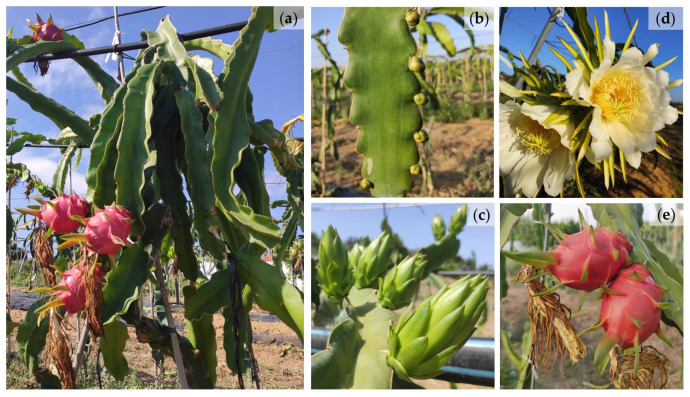
Plant of pitaya *Selenicereus undatus* (Haw.) D.R. Hunt (**a**), flower buds (**b**,**c**), flowers (**d**) and fruits (**e**).

The common name of these plants is different between countries and regions: *pitahaya* in Central America, “pitaya” or “dragon fruit” in North America and most European countries, “pitaia" in Portugal and Brazil, “fruta del dragon” or “pitaya” in Spain, “drachenfrucht” in Germany, “fruit du dragon” in France, “huǒ lóng guǒ” in China, “thanh long” in Vietnam, “φρούτο του δράκου” in Greece, “frutto del drago” or “pitaya” in Italy, and “vine cacti” in Israel [21,22]. The name pitaya is also attributed to a vast number of species of Cactaceae. Some authors use “pitaya” for almost all columnar and epiphytic cacti that produce edible fruits while others restrict this term only to the epiphytic cacti. Although in many cases the terms “pitaya” and “dragon fruit” are considered synonymous, the latter term is applied only to cultivars which produce fruits with long bracts, belonging to the genus *Selenicereus* (Figure 2).

There are other species of fruit-producing cactus, also with economic importance, such as the “pitaya de mayo”, “pitaya de junio”, “chirinola” or “cardón dato”, that belong to the genera *Cereus* Mill. and *Stenocereus* (A. Berger) Riccob. They have columnar stems that can reach up to 4 m in height without any kind of support [23,24] and very prickly fruits rarely reaching more than 400 g. These species either originate from the Caribbean region and northern South America [25] or are endemic to Mexico and the southern United States [26] and will not be discussed in this review. 

Hereafter, we will use the term “pitaya” only for the genus *Selenicereus* (A. Berger) Britton and Rose, which contains most cultivated species in Europe. Cacti of this genus can grow in the natural habitat as terrestrial, hemi-epiphytic, epiphytic or epipetric [27,28], but are cultivated as climbing terrestrial plants.

### 3.2. Species and Cultivars

The designation of pitaya species and cultivars tend to be used interchangeably, both in technical publications as in personal communications. This absence of a clear distinction between those designations is confusing, and makes it difficult to identify, not only the different plant species available in each country, but also cases of synonymy and homonymy and consequently warning us to the potential genetic proximity between cultivars.

There are four main pitaya species cultivated all over the world (Table 1): *S. undatus* (Haw.) D.R. Hunt, *S. monacanthus* (Lem.) D.R. Hunt (formerly named *Hylocereus monacanthus* (Lem.) Britton and Rose or *Hylocereus polyrhizus* (F.A.C. Weber) Britton and Rose), *S. costaricensis* (F.A.C. Weber) S. Arias and N. Korotkova ex Hammel. and *S. megalanthus* (K. Schum. ex Vaupel) Moran [6].

The species *S. undatus* produces fruits with red or yellow peel, white pulp and long bracts but it presents a great diversity of genotypes, with different agronomic characteristics and fruit quality [29,30]. The most important cultivars of this species (‘Common White’ and ‘Vietnamese White’) produce fruits with red peel and white pulp. The ‘Common White’ cultivar contains several clones that differ from each other in terms of shape (more or less rounded), fruit size and bract size. This cultivar is being replaced due to its self-incompatibility and tendency to form a gelatinous center, not appreciated by consumers. ‘Vietnamese White’ is probably the most cultivated cultivar in the Iberian Peninsula. It differs from the previous cultivar by having larger bracts and has the advantage of being self-compatible, at least, the clones cultivated in that region. The fruits of both cultivars are large, weighing between 500 and 1000 g. There are other cultivars of this species, with similar characteristics.

Although most cultivars of *S. undatus* have red peel, there are some cultivars of this species with yellow peel, namely, ‘Golden of Israel’ and ‘Golden Isis’, the last one from Australia. These two cultivars are very similar in morphology, but the fruit of ‘Golden of Israel’ is more elongated than that of ‘Golden Isis’. Both cultivars can be considered partially self-compatible, because when self-pollinated, they set fruit, although small (about 200 to 350 g) compared to those obtained by cross pollination (about 500 to 850 g). However, some producers consider ‘Golden Isis’ to be self-incompatible. More studies are needed to be sure about the differences between these two cultivars. Regarding sweetness, the value of total soluble solids content of all *S. undatus* cultivars is around 15 °Brix.

The short bracts and prickles of the fruit of *S. megalanthus* are the main characteristics that allow one to differentiate this species from the *S. undatus* yellow cultivars. The prickles come off easily when the fruit is ripe. *S. megalanthus* includes two main commercial cultivars: ‘Palora’, from Ecuador and ‘Colombian yellow’, from Colombia. There are also others like ‘Churuja’, ‘Golden Ball’, ‘Boliviana’ and ‘Amazonas’ from Peru, less cultivated than the previous ones. All of them are self-fertile and have an extended production season. The fruits are highly acceptable by consumers, having a high TSS content (22 °Brix), and a weight between 200 g and 500 g. However, their susceptibility to nematodes [31,32] and poor development at low altitudes are agronomic limitations requiring a more complex management in these conditions. 

The main cultivated species of red peel with red pulp are *S. costaricensis* and *S. monacanthus* (previously classified as *Hylocereus polyrhizus*). They have gained popularity among consumers due to their vibrant appearance, unique taste, and health benefits due to a high betalain content [33], generating more demand in the market. The species *S. costaricensis* is known worldwide as pitaya ‘Costa Rica’ and as ‘Roxa do Pará’ in Brazil. The species *S. monacanthus* is very popular in Asia and is distributed throughout the world. The most important cultivars of this species are ‘Tesoro’ (the most important in the Iberian Peninsula), ‘Boreal Red’, ‘Royal Red’, ‘Chinesa’ and ‘Taiwan Red’. These fully red fruits are medium to large sized, measuring 10 cm to 15 cm in length and weighing from 400 g to 1000 g. The color intensity of the pulp and peel can vary slightly between cultivars, from dark red and purple to bright pink. The sweetness level is around 18 °Brix [34].

There are currently some breeding programs underway that have been releasing new cultivars. Some of these new pitaya cultivars have better appearance and taste, are spineless, self-compatible and have shown long shelf life. EMBRAPA (Empresa Brasileira de Pesquisa Agropecuária) in Brazil carried out genetic improvement work, accompanied by validation in different regions of the country and production systems. This led to the registration of five new cultivars: ‘BRS Lua do Cerrado’ (*S. undatus*; red peel and white pulp), ‘BRS Luz do Cerrado’ (*S. undatus*; red peel and white pulp), ‘BRS Minipitaia do Cerrado’ (*S. setaceus*; red peel with prickles and white pulp), ‘BRS Granada do Cerrado’ (*S. undatus* × *S. costaricensis*; red peel and purple pulp) and ‘BRS Âmbar do Cerrado’ (*S. megalanthus*; yellow peel with prickles and white pulp) [35,36]. These cultivars were already imported into the Andalusian region of Spain and showed good development and production. 

In recent years, dozens of new cultivars have been obtained in North America: ‘DF 14’, ‘DF 16’, ‘N97-17’, ‘NOI-13’, ‘NOI-14’, ‘American Beauty’, ‘Bien Hoa Red’ (*S. guatemalensis*), ‘Armando’ (*S. monacanthus*), ‘Bien Hoa White’, ‘Mexicana’, ‘Seoul Kitchen’, ‘Vietnamese Giant’ (S. *undatus*), ‘Cebra’, ‘Lisa’, ‘Orejona’, ‘Rosa’, ‘San Ignacio’ (*S. monacanthus*), ‘Colombiana’ or ‘Yellow Dragon’ (*S. megalanthus*), ‘Delight’, ‘Halley’s Comet’, ‘Physical Graffiti’, ‘Sin Espinas’ (*Selenicereus* sp.), ‘El Grullo’, ‘Valdivia Roja’ (*S. ocamponis*) [9,37,38]. Some of them were imported and are being widely accepted in the Iberian Peninsula: ‘American Beauty’ (*S. guatemalensis*), ‘Delight’ and ‘Physical Graffiti’ (*S. guatemalensis* × *S. undatus*). The cultivars ‘Boreal Red’ and ‘Taiwan Red’, from Asia, are also gaining importance on farms in the Iberian Peninsula. 

**Figure 3:**
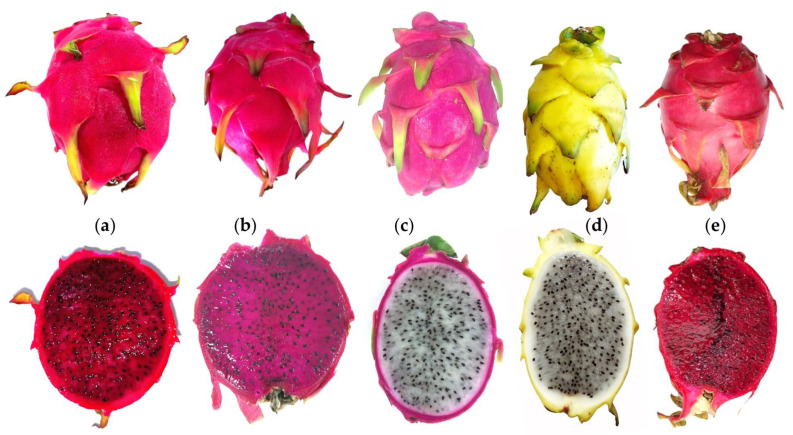
Fruits of the main species and cultivars cultivated in the Iberian Peninsula. *Selenicereus monacanthus* ‘Tesoro’ (**a**), *Selenicereus costaricensis* ‘Costa Rica’ (**b**), *Selenicereus undatus*. ‘Vietnamese White’ (**c**) and ‘Golden Yellow’ (**d**), and *Selenicereus monacanthus* × *Selenicereus undatus* ‘Hybridum’ (**e**).

Although many pitaya species and cultivars have been imported to the Iberian Peninsula in recent years, the most cultivated (with better acceptance by producers and consumers) so far are: *S. monacanthus* ‘Tesoro’, *S. costaricensis* ‘Costa Rica’, *S. undatus* ‘Vietinamese White’ and ‘Golden Yellow’ and *S. monacanthus* × *S. undatus* ‘Hybridum’ (Figure 3).

## 4. Edaphoclimatic Requirements

**Figure 4:**
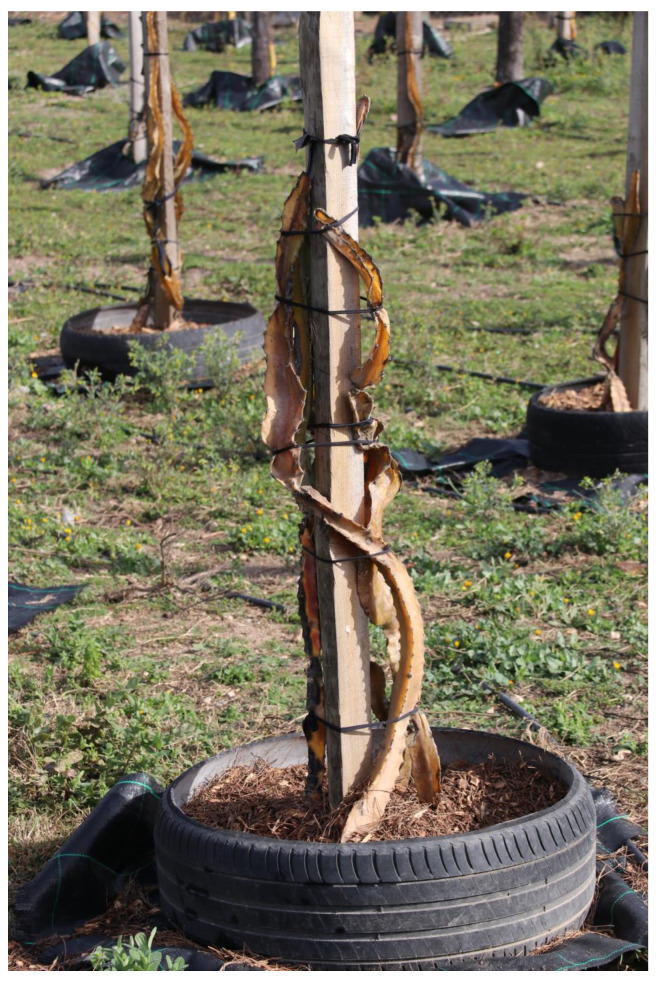
Frost damage in pitaya plantation in Algarve, in February 2019.

Pitaya develops in diverse edaphoclimatic conditions. Native to warm climates, pitaya develops better when temperature ranges from 18 °C to 26 °C, although it can be grown in regions with average temperatures of 14 °C to 32 °C. Above 28 °C, per each degree Celsius of temperature increase, plants of *S. undatus* and *S. megalanthus* show a 58% decrease in net CO_2_ uptake rates during the night phase, mediated by phosphoenolpyruvate carboxylase (PEPC) and a 30% decrease during the early morning phase, mediated by ribulose-1, 5-bisphosphate carboxylase/oxygenase (RuBisCO) [39]. Pitaya can withstand a maximum temperature of up to 40 °C [40], as long as the root system has access to water; however, temperatures above 38 °C inhibit flowering. In Mediterranean countries high temperatures are frequent in greenhouses during the summer, requiring measures of prevention like greenhouse painting or shade nets. However, frost is the greatest limitation for pitaya cultivation, even in greenhouse conditions. The minimum temperature pitaya can withstand is −2 °C, while lower temperatures as −4 °C causes plant death [41]. In the Iberian Peninsula, severe frost damage has been observed in open field (Figure 4). Therefore, to establish a pitaya plantation, it is necessary to make sure that the probability of frost is very low. 

Pitaya can grow in various types of soil, with preference for those with a high content of organic matter (which conserves moisture) well drained, and with a sandy loam texture. Pitaya can be cultivated in semi-arid climates, given its ability to withstand periods of drought [42,43]. The ideal soil pH for pitaya is between 5.5 and 6.5 [44], but it seems there is no restriction to development in alkaline soils, which are frequent in Mediterranean countries. Pitaya is sensitive to salinity, especially when associated with high levels of sodium [45,46]. 

Unlike desert cacti, pitaya is native to regions where rainfall is around 1730–2540 mm/year [47], although it can withstand rainfall between 500 and 700 mm/year. Excessive precipitation may cause floral abscission and fruit rot. Even so, in the Mediterranean region, floral abscission is not very common, since the rainy season does not coincide with the flowering period of pitaya. Fruit rot can occur in autumn, when the ripening of the last fruits can coincide with the first rains. On the other hand, in soils with a heavy texture, waterlogging can lead to cladode rot when in contact with the soil. To avoid these problems and prevent cases of excessive rainfall in field cultivation, the plants are installed on ridges or raised beds.

In its native environment, in tropical forests, pitaya grows in semi-shady conditions. This could be the reason why, in Southern Europe, pitayas develop better in shaded greenhouses. The species *S. monacanthus* grows better in 30% shade while *S. megalanthus* performance is better achieved in 60% shade [48]. The absence of a wax layer coating and sunken stomata in this later species is probably the reason for shade preference. Some authors report that the red pitaya *Selenicereus* spp. exposed to natural conditions of full sunlight in the hottest seasons of the year presents yellowish cladodes, while those protected by 35% shading reveal higher photosynthetic activity, better water use efficiency and greater vegetative growth [49,50]. However, observations made on three-year-old plants growing outdoors with maximum sun exposure did not show particularly yellow cladodes in conditions of maximum sun exposure. Sunburn often occurs when the plants are transplanted from a shady place to open ground [51].

## 5. Reproductive Biology

### 5.1. Flowering

As long as the plant grows in suitable conditions and depending on the state of maturation and development of the root system, flowering occurs after the first year of planting, from late spring to early autumn [52], being more intense in midsummer [53].

The flower bud develops for 25 to 35 days until anthesis occurs. During flowering period, new flower buds appear in various peaks, so different stages of development of both flowers and fruits are present in the same plant. The number of flowering peaks tends to increase with the age of the plant: three-year-old plants can emit more than five peaks during the summer, and even more in older plants. The intensity of flowering tends to be higher in well-managed plantations where good cultural practices such as fertilization, watering, and pruning are implemented.

The flowers are emitted in the axillary buds located on the edges of the cladodes, where the spines are also located. The flowers are large (15–30 cm), white, and hermaphrodite. The stamens are present in high quantity (up to 800). In some species, such as *S. undatus,* the stamens are shorter than the stigma, with differences in height of at least 2 cm. However, in other species, such as *S. megalanthus,* the anthers and the stigma are at the same height, facilitating self-pollination [54]. The ovary in plants of genus *Selenicereus* is inferior. 

Flowers open only at night for 8 to 12 h. In the south of Portugal, flowers start opening at 7 p.m. and at 11 p.m. are completely open. Pre-anthesis is characterized by an increase in the perianth’s volume followed by the opening of the sepals that starts in the afternoon. The flower opens at the beginning of the night. Anther dehiscence in flowers of *S. undatus* has been observed in the pre-anthesis phase, with flowers not yet fully open and since the stigma is located above the anthers, no natural pollination occurs [55,56]. 

The flower remains open until the next morning, even if it has not been fertilized and then closes and withers. Temperature and luminosity influence the opening and closing of the flower: on hot days, the flower may open earlier, around 6 p.m.; if temperature remains high, the flower withers earlier; if the day is cooler and cloudy, the flower can remain open until 11:00–12:00 a.m., although, generally, from 10 a.m. onwards, the flower is clearly wilted [57,58].

### 5.2. Pollination

Pollination occurs when pollen grains fall on the stigma and germinate, with a pollen tube emerging that grows inside the style towards the flower ovary. The more fertilized ovules, the more seeds will be formed, and bigger the fruit will be [59]. Two critical factors for the adhesion of pollen grains to the stigma and subsequent germination are essential for the success of pollination: stigma receptivity and pollen viability. Thus, pollination at the right moment of stigma receptivity ensures fertilization and fruit development [55]. Fruit development takes 30 to 35 days in summer, and this period is extended with lower temperatures in mid-autumn and early winter [60,61,62].

Pitaya flower pollination, for some cultivars, is associated with mechanisms of self-incompatibility. In this case, cross-pollination is necessary [63] and can be settled by manual pollination or through the action of natural pollinators as moths and bats [64] in regions where pitaya is native. In the southern regions of Portugal and Spain, bat species are insectivores [65,66,67]. Despite the scarce presence of nocturnal pollinators in the Iberian Peninsula, pollination occurs by the action of bees (*Apis mellifera*) mainly in the early hours of the day. These are the most abundant diurnal visitors to pitaya flowers, attracted by their strong odor [56,68]. The action of bees was also observed before dusk, which suggests the availability of pollen at that time. Here, the pollination of bees may even be more efficient since the circulation space for them (between entrances and exits of the flower) is smaller than when the flower is completely open, increasing the probability of them traveling through the stigma leaving pollen on it. Additionally, the greater the number of bees per flower, the greater the probability of brushing the pollen-covered body on the stigma, increasing the probability of pollination. The activity of bees depends on the weather conditions in the early morning, the density of hives in the plantation, the amount of open pitaya flowers and their distribution in the plantation and also the presence of other flowers that attract bees.

## 6. Genetics, Xenia and Metaxenia

In Cactaceae, the monoploid number is *x* = 11. Most *Selenicereus* species (especially those classified as *Hylocereus* by specialists in this subject) are diploid species, having 2*n* = 2*x* = 22 chromosomes, whereas *S*. *megalanthus* (syn. *H*. *megalanthus*) is a tetraploid species, having 2*n* = 4*x* = 44 chromosomes. This evidences the genetic proximity between all pitayas and justifies the possibility of performing interspecific homoploid crosses (2*n* × 2*n*) to obtain diploid hybrids [11,69,70].

In Israel, since 1984, studies have been carried out on dragon fruit genetics and the current state of the art has been reported [11]. The research revealed self-compatibility in wild tetraploid *S. megalanthus*, as well as in induced autotetraploid *S. monacanthus* and in artificial allotetraploids [11]. The same research team found cases of self-incompatibility in *S*. *monacanthus* (referred to as *H*. *polyrhizus*) and a weakened incompatibility reaction in *S. undatus* and *S. megalanthus* [69]. That research team from Israel has already produced new cultivars that may be interesting for the Iberian Peninsula and has developed knowledge that can be used by other researchers in dragon fruit breeding programs.

The effect of the pollen grain (male parental) on embryo tissue is known as xenia. Metaxenia is a phenomenon that occurs when the pollen from one plant affects the characteristics of the fruit produced by another plant. The boundary between the two terms is not always clear, as some authors use the term xenia in a broader sense, including metaxenia [71,72]. Metaxenia can support the use of pollination as an effective technique to improve fruit quality [73]. If pollen from another species or cultivar is compatible and capable of fertilizing the ovules, the metaxenia effect can result in differences in seed and fruit characteristics compared to what would be expected if self-pollination occurred [74]. 

The use of metaxenia in fruit production is not unique to pitaya, and it has been tested and observed in other plants such as citrus [75,76], apple [77] and blueberry [78]. Metaxenia can lead to changes in the shape, size, color and flavor of the fruit. The genetic characterization of pitaya has been studied to some extent [79]; however, there is still much more to understand to clarify the metaxenia effect.

The nutritional content of pitaya fruit is influenced by the genetic characteristics of the plant. In fact, several studies describe distinct antioxidant and physicochemical attributes of pitaya fruits depending on the species and cultivar [80,81,82]. Furthermore, one way to lengthen the marketing season could be to pollinate the flowers with pollen from another species that affects the ripening time of the fruit. A delay in ripening can enhance the accumulation of soluble solids, resulting in sweeter fruits, in addition to harvesting fruits outside peak supply to obtain a better selling price [83].

The Pitaya Genome and Multiomics Database (PGMD) (http://www.pitayagenomic.com, accessed on 22 March 2023) integrates data on genetic variability, gene expression, miRNA profiles, metabolite and proteomic data from distinct plant tissues and from fruit developmental stages of several pitaya cultivars. It also provides tools for online analysis and visualization of new findings from phenotypic datasets, including transcriptomic and metabolite data [84].

Further research in this area may unveil the functional characteristics, nutritional content, shelf life, fruit size and productivity of pitaya affected by metaxenia.

## 7. Physiology

Pitaya belongs to the Cactaceae family and, as such, has Crassulacean Acid Metabolism (CAM), which is one of the main carbon-concentrating mechanisms that influences the behavior of the stomata: they open at night and close during the day. The carbon dioxide absorbed during the night is fixed in the form of malic acid, which is subsequently decarboxylated [59]. According to Nobel and De la Barrera [85], *S. undatus* CO_2_ uptake, under wet conditions, was found to be 232 mmol m^−2^ day^−1^ and water loss 53 mmol m^−2^ day^−1^, which resulted in a water efficiency of 0.0044 CO_2_/H_2_O. This value is three to four times higher than those of C_3_ or C_4_ plants grown under the same conditions [86], which demonstrates the adaptation of pitaya to environments of low water availability, unfortunately increasingly frequent in the Mediterranean Basin. When drought lasts for ten days, the net CO_2_ uptake decreases by 57% and the maximal rate of net CO_2_ uptake occurs during the night, when the temperatures are lower and transpiration is also lower. Under drought conditions there is a reduction in stomatal conductance of water and stomata closes, a response attributed to the abscisic acid produced in the roots. In fact, there is a delay on the CO_2_ uptake inhibition when *S. undatus* roots are cut, showing the importance of this hormone on water use by the plant [85]. Another metabolic process described in *S. undatus* by Nobel [87] is the transferring of water from the leaf parenchyma to the chlorenchyma maintaining a net CO_2_ uptake under drought conditions.

A higher net CO_2_ uptake at night indicates that night temperature is more important for photosynthesis than day temperature. The maximal CO_2_ uptake in *S. undatus* occurs when the combination of day/night temperatures is 30 °C/20 °C. Combinations of lower (18 °C/8 °C) or higher (38 °C/28 °C) temperatures lead to a 50% reduction in net CO_2_ absorption. With day/night temperatures of 42 °C/32 °C, respectively, the daily net CO_2_ uptake reaches zero [85].

An increase in atmospheric CO_2_ concentration stimulates photosynthesis and, consequently, vegetative and reproductive growth [88,89]. Therefore, CO_2_ enrichment can be used to increase productivity under greenhouse conditions (prevalent in the Iberian Peninsula).

Under moderate drought conditions, around 50% of field water capacity (FWC), an increase was detected in the toxic compound malonaldehyde and of superoxide dismutase and peroxidase enzymes, which are able to protect the cells and maintain the water needs by osmotic adjustment with proline [43]. However, in case of severe drought, less than 40% FWC, this system seems to fail. Even in regions with abundant rain, irrigation should be recommended as the largest biomass of *Selenicereus* spp. is obtained with 70–80% FWC, while at values of 50–60% FWC, the biomass is allocated in roots, reducing the fruit production potential [43].

If a drought situation lasts for 10 days, CO_2_ uptake is reduced by 57% with the help of stomata closure. This mechanism gives the pitaya enormous adaptability to adverse environments and ability to survive in environmental conditions that would be fatal to other fruit species, tolerating severe drought [42]. These particularities are also due to other adaptation mechanisms, such as the ability to accumulate water in the stem, the absence of leaves and, in some varieties, the presence of a waxy layer on the stem. These characteristics enable pitayas to adapt their physiology to environmental conditions and explain its worldwide geographical distribution. Thus, pitaya develops well in hot and humid climates, as well as in dry environments. In a scenario of climate change, specifically in regions with increasing prolonged periods of drought, CAM metabolism crops may be an interesting option for fruit production.

## 8. Nutritional Characteristics and Uses

In addition to its exotic and extremely attractive appearance, pitaya fruit has been sought-after, not only for its bioactive compounds, such as vitamins, phenolic compounds, and pigments such as betalains, which act in the human body as antioxidants, but also for accelerating metabolism, converting nutrients into energy and reducing fat deposits [90,91,92,93]. 

With just 50 calories in 100 g of fresh fruit, pitaya is recognized as a low-calorie fruit, a very advantageous feature when the demand for healthy foods is increasing. The fruit is also a recognized source of lipids, fibers, carbohydrates, vitamin C, calcium, iron, phosphorus and potassium. These fruit properties help in protection against diabetes type 2 by reducing blood glucose and in colon cancer prevention. It can also reduce bacterial infections and its seed contains around 50% of essential fatty oils (omega 3 and 6) that may help to reduce the risk of cardiovascular diseases [94,95,96]. Additionally, the oil from pitaya seeds has an expressive content of natural antioxidants and tocopherols; thus, it can be used as a source of edible oil or incorporated into cosmetics or drugs [97,98,99]. Furthermore, the fruit has a high potential for consumption in processed products.

The flower buds can also be consumed. They may be grilled or fried or used in soups or salads. The flowers, when dried after the fruit sets, can be used for infusions. 

Pitaya fruits are also an interesting option for obtaining natural dyes, whose main advantages when compared to synthetic dyes are the absence of toxicity, the renewability, and lack of evidence of health risks [100,101]. For this purpose, the peel of the fruit is cited by several studies as a usable residue in the production of natural dyes [102,103,104,105]. Red or purple pigments from pitaya species such as *S. monacanthus* and *S. costaricensis* are potential sources of dyes for the food industry [106] stable during processing and storage, thus representing one more option to the use of pitaya [107,108].

## 9. Previous Reviews and Published Scientific Articles

**Figure 5:**
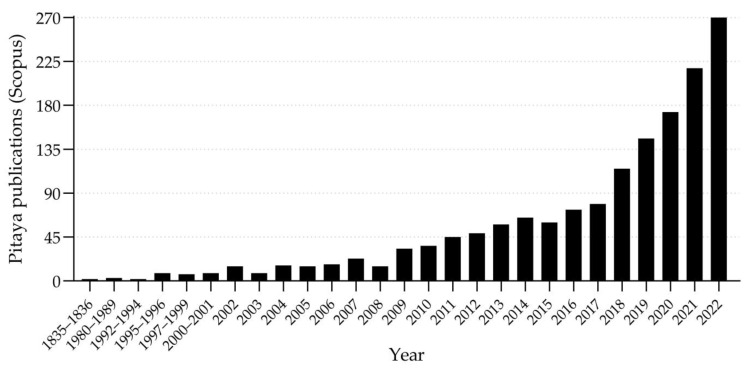
Number of scientific articles on pitaya, published until the year 2022 in journals indexed by the Scopus platform. Articles containing the words «dragon fruit» or «pitaya» or «*Hylocereus*» or «*Selenicereus*» in the title or keywords were considered.

Pitaya is a relatively new crop in Iberian Peninsula that has been growing all over the world in recent years, both in the production and consumption of the fruit, as well as in scientific research. A search was carried out on one of the main indexing platforms for articles with a worldwide impact factor (https://www.scopus.com, accessed on 1 April 2023) and a total of 1600 publications were found by the year 2022 (Figure 5), with considerable increase from 2009 and exponential increase in the last five years.

In addition to publications of scientific articles on pitayas with original data, several scientific reviews on this crop have also been published, mainly in the last decade (Table 2). Research on pitaya covers a wide range of topics, including its nutritional composition, antioxidant properties, anti-inflammatory effects and potential therapeutic applications. Among the most recent publications, the majority have focused on the characterization of pitaya bioactive compounds, such as flavonoids, betacyanins and polysaccharides, and their potential health benefits. In addition, there are also publications on post-harvest handling and processing of pitaya to improve its shelf life and preserve its quality. Other studies analyzed the potential use of pitaya as a natural dye, functional ingredient in food products and as a source of bioactive compounds for nutraceuticals and pharmaceuticals, with the smallest portion focusing on agricultural sciences and pitaya phytotechnics.

It is extremely important and necessary to carry out scientific research focused on field practice in the cultivation of pitaya in the Iberian Peninsula and around the world, aiming at the effective cultivation of varieties with great productive potential and good acceptance of the fruits by consumers.

## 10. Future and Conclusions

Considering the effects of global warming on climate change and on natural resources, pitaya’s relative low water requirement and the economic profit have drawn attention to its cultivation. Thus, pitaya is a crop whose cultivation has great potential to meet the concerns of agricultural activity and consumer demands for foods with high nutritional value.

Considering the climatic conditions of the Iberian Peninsula, using appropriate agronomic techniques, pitaya cultivation can achieve high productivity. The main applicability of the pitaya is the consumption of fresh fruit and its potential to be processed. Other parts of the plant, such as the flower or the cladode itself, can be used for human and animal food consumption, or even in the pharmaceutical or cosmetic industries. Therefore, pitaya constitutes a viable crop option that justifies the continuity of research work on its cultivation.

## Figures and Tables

**Table 1:** Main characteristics of pitaya species and their cultivars present in the Iberian Peninsula.

Species and Cultivars	Peel Color	Pulp Color	Fruit Size (g)	TSS * (°Brix)	Fertility **
*S. undatus*					
‘Common White’	Red	White	500–1000	15	SI
‘Vietnamese White’	Red	White	500–1000	15	SF
‘Golden’	Yellow	White	200–800	15	PSC
*S. monacanthus*					
‘Tesoro’	Red	Red	400–800	18	SC
*S. costaricensis*					
‘Costa Rica’	Red	Red	400–800	18	SC
*S. megalanthus*					
‘Palora’	Yellow	White	200–500	22	SC
‘Colombian yellow’	Yellow	White	200–500	22	SC

* TSS: Total Soluble Solids (average value); ** SC: self-compatible; SI: self-incompatible; PSI: partially self-compatible.

**Table 2:** Reviews published on pitaya in peer-reviewed journals indexed by the main database platforms worldwide.

Topic	Amount	References
Agricultural sciences	6	[6,68,109,110,111,112]
Food chemistry and health	16	[68,90,91,94,100,113,114,115,116,117,118,119,120,121,122,123]

Articles containing the words «review», «dragon fruit», «pitaya», «*Hylocereus*» or «*Selenicereus*» in the title or keywords were considered.

## Data Availability

Not applicable.
